# Comparison of Microbiota in Patients Treated by Surgery or Chemotherapy by 16S rRNA Sequencing Reveals Potential Biomarkers for Colorectal Cancer Therapy

**DOI:** 10.3389/fmicb.2018.01607

**Published:** 2018-07-17

**Authors:** Xingming Deng, Zhuofei Li, Guan Li, Bei Li, Xinhan Jin, Guoqing Lyu

**Affiliations:** Department of Gastrointestinal Surgery, Peking University Shenzhen Hospital, Shenzhen, China

**Keywords:** bacterial diversity, chemotherapy, colorectal cancer, surgery, 16S rRNA sequencing

## Abstract

Colorectal cancer (CRC) is the third most diagnosed cancer worldwide due to its high difficulty in early diagnosis, high mortality rate and short life span. Recent publications have demonstrated the involvement of the commensal gut microbiota in the initiation, progression and chemoresistance of CRC. However, this microbial community has not been explored within CRC patients after anti-cancer treatments. To this end, we performed next generation sequencing-based metagenomic analysis to determine the composition of the microbiota in CRC patients after anti-cancer treatments. The microbial 16S rRNA genes were analyzed from a total of 69 fecal samples from four clinical groups, including healthy individuals, CRC patients, and CRC patients treated with surgery or chemotherapy. The findings suggested that surgery greatly reduced the bacterial diversity of the microbiota in CRC patients. Moreover, *Fusobacterium nucleatum* were shown to confer chemoresistance during CRC therapy, and certain bacterial strains or genera, such as the genus *Sutterella* and species *Veillonella dispar*, were specifically associated with CRC patients who were treated with chemotherapeutic cocktails, suggesting their potential relationships with chemoresistance. These candidate bacterial genera or strains may have the ability to enhance the dosage response to conventional chemotherapeutic cocktails or reduce the side effects of these cocktails. A combination of common CRC risk factors, such as age, gender and BMI, identified in this study improved our understanding of the microbial community and its compositional variation during anti-cancer treatments. However, the underlying mechanisms of these microbial candidates remain to be investigated in animal models. Taken together, the findings of this study indicate that fecal microbiome-based approaches may provide additional methods for monitoring and optimizing anti-cancer treatments.

## Introduction

Colorectal cancer (CRC) is considered the third most frequently diagnosed cancer in the world and leads to high mortality (Jemal, 2011; Ferlay et al., 2013). Generally, over 1 million people are affected due to various intrinsic and environmental factors. For instance, lifestyle and dietary risks, such as drinking problems, excessive consumption of red meat in the diet and long-term intestinal inflammation, greatly increase the possibility of tumor initiation in the gastrointestinal tract (Larsson et al., 2005; Nugent et al., 2005; Huxley et al., 2009).

Genetic studies indicate that the loss of tumor suppressor genes and mutation activation/inactivation may contribute to the development of CRC (Vogelstein and Kinzler, 2004; Brenner et al., 2014). Recent advancements in CRC research suggest that variations in the composition of microorganisms (i.e., gut microbiome) in the gastrointestinal tract may play crucial roles in CRC initiation and development (Cheesman et al., 2011). In general, approximately 10^3^ commensal bacteria species can be counted in the healthy human intestinal tract and are essential for human health by promoting food digestion and intestinal epithelial homeostasis under normal conditions (Hooper and Gordon, 2001). The physiological and molecular mechanisms of the gut microbiome and its association with CRC have been extensively studied over the past decade (Dolara et al., 2002; Stappenbeck et al., 2002; Rakoff-Nahoum and Medzhitov, 2007). Initial evidence linking CRC to microorganisms has demonstrated that larger tumor sizes are observed in mice kept under specific pathogen-free conditions (Dove et al., 1997). In addition, *Streptococcus bovis* and *Clostridium septicum* have been identified to be associated with clinical CRC samples via culture-based analysis (Boleij et al., 2009; Seder et al., 2009). However, evidence from case reports is not strong enough to draw conclusions. Hypotheses and research models have been established to indicate that the signature of the gut microbiome may affect tumor growth in the colon via direct or indirect mechanisms (Chen et al., 2012, 2013; Kostic et al., 2012). For example, the diet has been considered to be an important environmental factor that promotes adenoma in CRC patients by affecting the GI microbial composition (Chen et al., 2013). Different bacterial structures have been demonstrated to be associated with cancerous and non-cancerous tissue (Chen et al., 2012). With the development of next-generation of sequencing approaches, a number of studies have characterized the compositional changes of the microbiome between healthy individuals and CRC patients at different stages (Larsson et al., 2005; Rakoff-Nahoum and Medzhitov, 2007; Huxley et al., 2009). This microbial dysbiosis has been observed in several sample types, including the colorectal mucosa, tumor tissue and human feces (Sobhani et al., 2011; Geng et al., 2013). Bacterial species have been commonly identified across CRC samples (Wang et al., 2011; Zackular et al., 2014), but their roles in CRC development remain to be elucidated. For example, *Bacteroides fragilis* and some strains of *Escherichia coli* are positively correlated with colorectal tumor size by producing toxins to human intestinal cells (Costello et al., 2009). By contrast, bacterial strains with anti-tumor potential have been proposed as well. As metabolic products of anti-cancer microbes, short-chain fatty acids (SCFA), such as butyrate, inhibit tumor growth in the colon (Boon et al., 2002). Recent has shown that the symbiotic microorganisms in the human intestinal tract are able to affect the local tissue metabolic activity and development of host organisms (Lee and Mazmanian, 2010). Furthermore, commensal bacteria have the ability to influence the inflammation process and immune responses of the host organism systemically (Ichinohe et al., 2011; Clemente et al., 2012; McAleer and Kolls, 2012). For instance, commensal microbiota affect virus-specific antibody production during influenza virus infection (Ichinohe et al., 2011). In addition, commensal bacteria are able to promote the recognition of viruses by antigen-presenting cells (McAleer and Kolls, 2012). Thus, CRC initiation and development are regarded as a co-occurrence that leads to the establishment of tumor-promoting bacteria and elimination of anti-tumor bacteria.

The development of CRC is a stepwise process by which locally attached adenoma tissue initiates in the colon and progresses to invasive and metastatic carcinoma tissues over time (Fearon, 2011). Importantly, the chance of initiating carcinoma development can be largely reduced if early adenomas (stage I and II) are detected and surgically removed in time (Bernard Levin et al., 2008). Over 90% of CRC patients survive if the diagnosis occurs while the disease is still localized. However, there is a dramatic decrease of the survival percentage among CRC patients with later stage carcinomas. In addition to surgery, chemotherapy is usually used to inhibit tumor growth and cancer development in advanced CRC patients when metastasis to secondary organs occurs (Yu T. et al., 2017). Generally, chemicals with a broad-spectrum of cytotoxicity, such as capecitabine, 5-fluorouracil (5-FU) and oxaliplatin, are commonly used in combination for chemotherapy (Cartwright, 2012; Yu T. et al., 2017). As a first-line treatment of CRC, oxaliplatin is usually given in combination with 5-FU, which is known as the FOLFOX regimen (Wang and Li, 2012). These drugs can either effectively inhibit DNA replication of cancer cells (capecitabine and 5-FU) or arrest the cell cycle (oxaliplatin) (Walko and Lindley, 2005; Kelland, 2007). However, the survival rate of advanced CRC patients receiving the FOLFOX regimen is generally lower than 10% because of the chemoresistance of tumor tissues (Dahan et al., 2009). Studies indicate that novel immune checkpoint therapy barely has any effect these patients with stage III or IV CRC (Zou et al., 2016). Therefore, developing a better understanding of chemoresistance and its underlying mechanisms in CRC are current priorities. Emerging evidence has sown that there are links between the gut microbiota and chemotherapy (Iida et al., 2013). Previously identified CRC biomarkers, denoted as *Fusobacterium nucleatum*, have been characterized by multiple studies (Castellarin et al., 2012; Kostic et al., 2012). This species has been observed to be high abundance in adenoma tissues and progressively increases in richness during colorectal carcinogenesis (Castellarin et al., 2012; Kostic et al., 2012). Previous observations suggest that this species has the ability to promote carcinogenesis (Rubinstein et al., 2013) and has been detected to be co-localized with CRC epithelial cells (Abed et al., 2016). Recently, the mechanism of *Fusobacterium nucleatum* in CRC chemoresistance has been elucidated (Yu T. et al., 2017), providing a valuable target for the development of new chemotherapeutic methods for CRC treatment.

Although various studies have been performed to profile the microbial composition of the microbiota of CRC patients at different tumor developmental stages to assess its diagnostic potential, no metagenomic studies of the microbiota of CRC patients after surgery and chemotherapy have been reported. In this study, we hypothesized that using alternative biomarkers from the CRC-related microbiota could improve the probability of identify candidate microbes that are specifically associated with CRC patients treated by surgical debulking or chemotherapy. We performed standard 16S rRNA sequencing and bioinformatic analysis of the stool microbiome of healthy individuals, CRC patients, and CRC patients after either surgical debulking or chemotherapy. High-resolution images of the human CRC microbiome, especially those after patients underwent surgery or chemotherapy, were compared for potential biomarker identification. The results indicate that metagenomic analysis of the microbial composition is a valuable tool for biomarker identification and candidate selection for further investigation.

## Results

### Characteristics of the Sequencing Results

The experimental design and analytical pipeline are shown in **Supplementary Figure S1**. In general, a total of 69 samples were subjected to 16S rRNA sequencing. These samples were divided into four groups (**Table 1**) that included 33 healthy individuals (denoted as group C), 17 CRC patients (denoted as group I), 14 chemotherapy-treated CRC patients (denoted as group D) and 5 surgically treated CRC (denoted as group IO). In general, an average of 1259 operational taxonomic unit (OTUs) were identified across these 69 samples, with a minimum of 80 OTUs and a maximum of 2324 OTUs. The parameters, such as the dilution curve, Shannon curve, and rank abundance of OTUs, were evaluated to confirm the reliability of the sequencing data (**Supplementary Figure S2**).

**Table 1:** Characteristics of subjects in each clinical group.

	C	I	IO	D	
Age	48.3 (±19.1)	58.4 (±12.4)	53.0 (±9.0)	49.5 (±11.9)	Mean (*SD*)
BMI	22.2 (±2.8)	22.8 (±3.7)	23.6 (±4.9)	20.9 (±2.9)	Mean (*SD*)
Men	28	11	3	7	
Women	5	6	2	7	
Total	33	17	5	14	

### Differential Microbial Structure Among 69 Tested Samples

**FIGURE 1:**
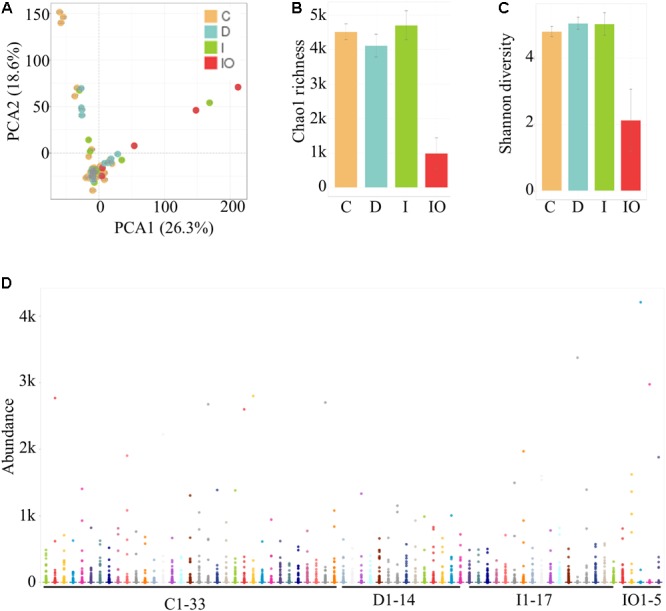
Identification and quantification of OTUs among 69 samples in this study. **(A)** PCA for all samples. **(B)** Representation of the microbiota richness **(B)** and diversity **(C)** of four clinical groups. C, Healthy individuals; D, CRC patients after chemotherapy; I, CRC patients before treatment; IO, CRC patients after surgical debulking. **(D)** Distribution of the microbial abundance for all samples.

**FIGURE 2:**
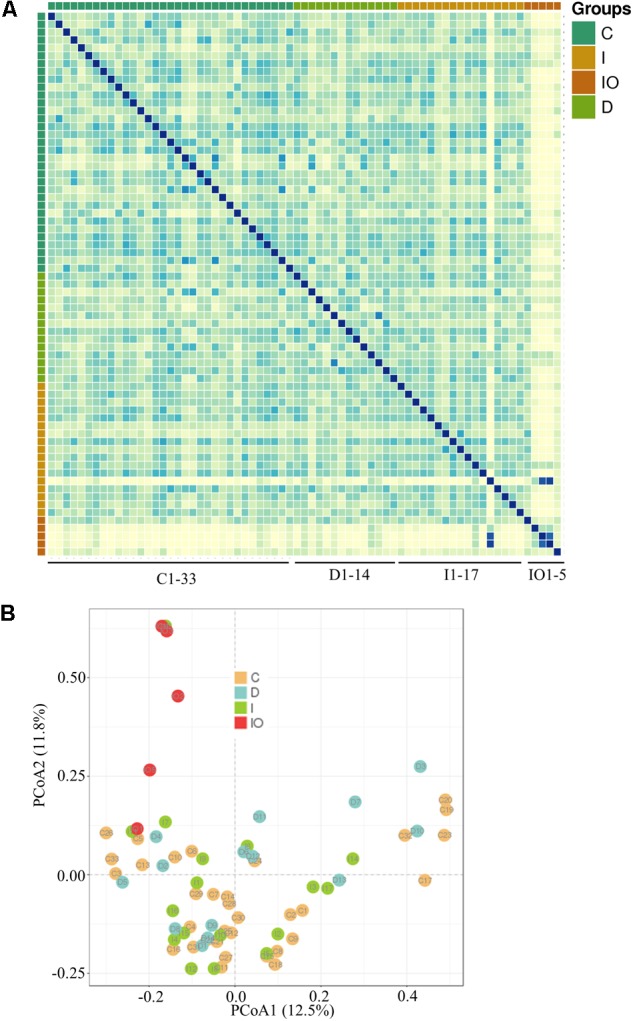
Representation of microbial beta-diversity. Heatmap representation **(A)** and PCoA **(B)** based on Brey-Curtis similarity.

**FIGURE 3:**
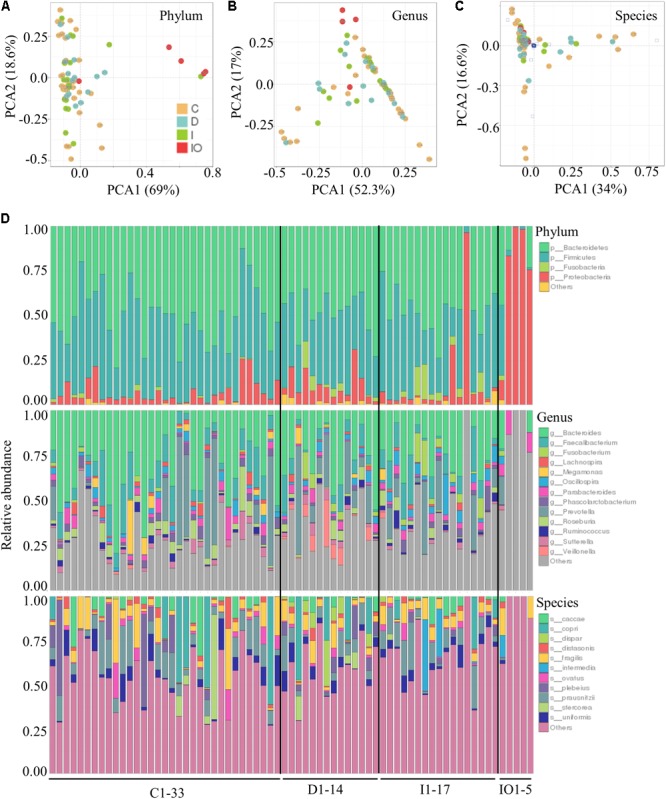
Relative abundance of the bacterial phyla, genera and species in each clinical group. PCoA at the phyla **(A)**, genera **(B)**, and species **(C)** levels for all clinical groups. **(D)** Relative abundance of the annotated OTUs for all of the samples tested in this study.

Stool samples were analyzed from the above mentioned four sampling groups (**Supplementary Figure S1**). The microbial composition of each sample showed no apparent variation according to principle component analysis (PCA) (**Figure 1A**). The Chao value indicated that three groups (C, D, and I) had higher community richness than that of group IO (**Figure 1B** and **Supplementary Table S1**). Similar patterns were observed regarding the Shannon diversity indicator (**Figure 1C**), suggesting that surgery greatly affected the structure of the microbiota in CRC patients. Subsequent statistical analysis using PERMANOVA determined considerable variations in microbial community structures among the four clinical groups (*P* = 0.001). In addition, large variations in both the number of OTUs and counts detected among all 69 samples indicated that considerable variations among individuals within each group and statistical analysis were required for subsequent group comparisons (**Figure 1D**). Abundance summaries showed that some OTUs were much higher in abundance than other OTUs among all samples (**Figure 1D**). Subsequently, both heatmap and PCoA calculated by Brey-Curtis similarity suggested that considerable variations exist among individual samples referred by beta-diversity (**Figure 2**). The high abundance OTUs provided interesting candidates for further analysis. Moreover, the microbiota structure of each sample was analyzed and presented at the phylum, genus and species levels (**Figure 3**). PCoA suggested that most of the IO group members were significantly different from those of the other three groups at the phylum level (**Figure 3A**), implying its unique microbiota composition. Among the phyla, *Bacteroidetes* and *Firmicutes* were the most abundant phyla in the C, I, and D groups, whereas the phylum *Proteobacteria* was found at high richness in the IO group (**Figure 3D**), indicating its specific association with CRC patients after surgery. By contrast, at both the genus and species levels, no further variations were observed among the four groups via PCoA (**Figures 3B,C**), but variations of the microbial composition in an individual genus or species were detected among all samples (**Figure 3D**).

### Detection of Microbial Groups Is Linked to Colorectal Cancer

Common microbial species detected in cancer studies (Chen et al., 2012; Feng et al., 2015) were repeatedly found in this batch analysis of CRC patients. For example, the phylum *Fusobacteria* was enriched in CRC patients (group I) in comparison to that in healthy individuals (group C). Furthermore, the genera *Fusobacterium, Oscillospira*, and *Prevotella* were all detected in CRC patients (group I) and CRC patients after chemotherapy (group D). The variations of the microbial composition after surgery and chemotherapy were further compared in subsequent analyses.

### Surgery Leads to a Unique Microbial Composition in CRC Patients

**FIGURE 4:**
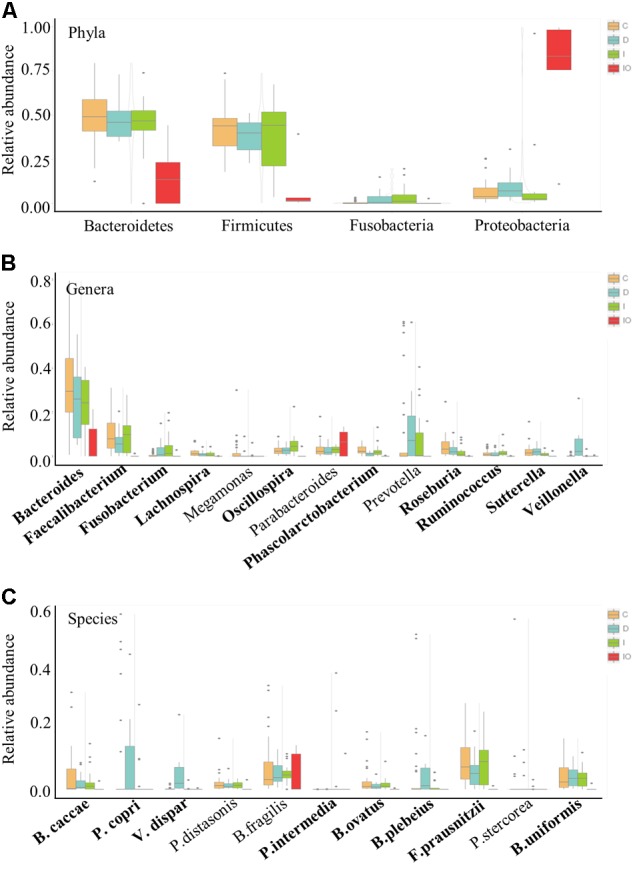
Relative abundance of the representative phyla, genera and species for group comparison. Representative high abundance microbes for all of the clinical groups in this study at the phyla **(A)**, genera **(B)**, and species **(C)** levels. C, healthy individuals; D, CRC patients after chemotherapy; I, CRC patients before treatment; IO, CRC patients after surgical debulking.

To further understand the differential microbiota structures among the four groups, microorganisms with a high relative abundance (>1%) were selected for comparison at the phylum, genus and species levels (**Figure 4**). Consistently, the majority of high abundance microbes present in group C, D, and I were not detected in the IO group. In comparison to the other three groups, the phyla *Bacteroidetes* and *Firmicutes* were much less abundant in the IO group. Meanwhile, a higher abundance of *Proteobacteria* was detected in the IO group than in the other groups (**Figure 4A**). Correspondingly, in the IO group, the genus *Bacteroides* was found at a lower relative abundance in comparison with that in the other three groups. This phenomenon indicates that surgical operation may greatly reduce microbial diversity and richness in CRC patients. However, the underlying mechanism remains to be elucidated.

### Specific Groups of Microbes Are Associated With Chemotherapy

Chemotherapy by drug cocktails is a routine method used in patients with advanced CRC. Here, we demonstrated that some microbial groups were tightly associated with CRC patients undergoing chemotherapy that included a combination of oxaliplatin and tegafur (a precursor of 5′-FU). At the genus level, *Veillonella* was uniquely present in group D patients (**Figure 4B**). In particular, *V. dispar* was only observed in chemotherapy-treated patients, but not in patients in the other three groups (**Figure 4C** and **Supplementary Figure S3**). Additionally, although not detected at the genus level, two other species, *Prevotella copri* and *Bacteroides plebeius*, were only enriched in patients treated by chemotherapy (**Figure 4C** and **Supplementary Figure S3**).

### Potential Biomarkers for CRC Patients Treated by Surgery or Chemotherapy

**FIGURE 5:**
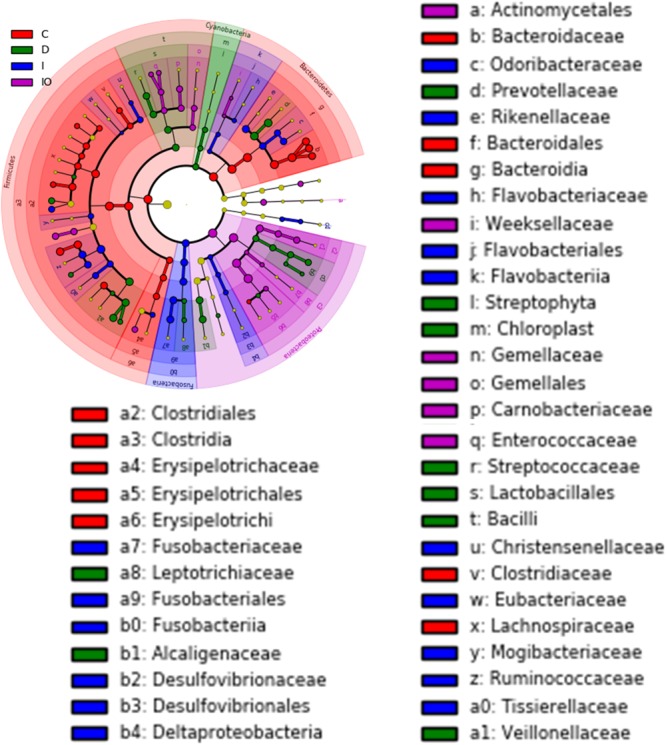
Cladogram view of the representative microbial structure among the four groups. Phylogenetic view of the representative microbiota for each clinical group. Dominant microbial classes for the specific groups are represented by different colors. C, healthy individuals; D, CRC patients after chemotherapy; I, CRC patients before treatment; IO, CRC patients after surgical debulking. The diameter of each dot is proportional to the OTU abundance.

**FIGURE 6:**
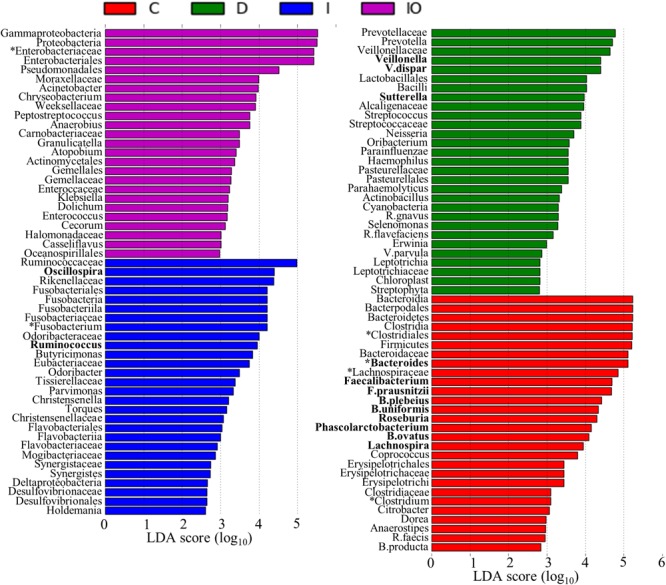
Histogram representation of potential biomarkers. Potential biomarkers for each clinical group are presented by the LDA scores from the phylum to species. C, healthy individuals; D, CRC patients after chemotherapy; I, CRC patients before treatment; IO, CRC patients after surgical debulking.

**FIGURE 7:**
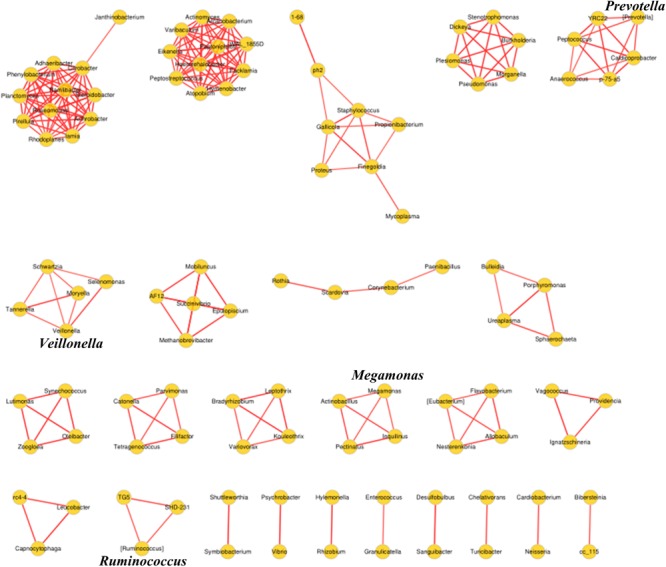
Association network diagram of bacterial genera among CRC patients from all the clinical groups. Network analysis conducted by spearman correlation (*R* > 0.8, *P* < 0.05, Red line, positive correlation) is shown.

Biomarkers are valuable targets for disease diagnosis and treatment improvement. Potential biomarkers (from phylum to species) for each group were identified using the LDA score (**Figures 5, 6**). As previously described, *Proteobacteria* was regarded as a biomarker in CRC patients treated by surgery. Moreover, *Fusobacteria* was considered a potential biomarker for CRC patients in this study, whereas *Cyanobacteria* was associated with CRC patients after chemotherapy (**Figure 5**). At the species level, species such as *V. dispar, F. prausnitzii, B. plebeius, B. ovatus*, and *B. uniformi* were repeatedly observed to have high LDA scores (**Figure 6**), suggesting their potential value as novel biomarkers for monitoring CRC patients after surgery or chemotherapy. In addition, network associations of bacterial structure identified among all the clinical groups was drafted (**Figure 7**). Several abundant bacterial genera (**Figure 4B**) such as *Prevotella, Veillonella, Megamonas*, and *Ruminococcus*, exhibited multiple positive links to other bacterial genera (*P* < 0.05, Spearman *R* > 0.8), suggesting their potential synergistic roles related to CRC.

## Discussion

### Comparison of Microbiome Changes Among the Four Tested Groups

Colorectal cancer (CRC) is considered to be a global public health problem since it has a high incidence and percentage of mortality and low diagnostic rate at early stages. Although a number of oncogenes related to CRC have been reported, the influence of additional environmental factors in CRC, such as the microbiome, is unknown. Previous metagenomic and transcriptomic studies have led to the development of novel specific and sensitive biomarkers for the diagnosis of CRC at the earliest stages (Mármol et al., 2017). In this study, considerable microbiome differences among healthy people (group C), CRC patients (group I), and CRC patients after surgery (group IO) or chemotherapy (group D) were observed by using PERMANOVA (*P* = 0.001), implying their potential roles in response to anti-cancer treatments. Previous studies have listed a number of microbial species that have been found in CRC patients (Chen et al., 2012; Zackular et al., 2013). Some of these species were present in our dataset as well. For example, *Fusobacterium* spp. were previously reported to co-exist with tumors and may positively regulate tumor cell propagation (Kostic et al., 2012; Rubinstein et al., 2013). In this study, the genus *Fusobacterium* was found to be enriched in both CRC patients (group I) and CRC patients after chemotherapy (group D), but depleted in healthy individuals and CRC patients after surgery (group IO, **Figure 4B**); therefore, the genus *Fusobacterium* was identified as a putative biomarker for CRC patients (**Figure 6**). This result suggested that *Fusobacterium* spp. were associated by CRC, but not by conventional chemotherapy. The abundance of *Fusobacterium* was largely reduced after surgical debulking, suggesting that there were potential differences between these two anti-cancer therapeutic approaches. In addition, *Bacteroides fragilis* has been considered to be a pathogenic species that is associated with colorectal tumor development (Sears et al., 2008). Previous reports indicated that carcinoma tissues had elevated levels of *B. fragilis* in comparison to early stage adenomas (Zackular et al., 2014), suggesting that this species maybe associated with tumor progression. However, no differences in *B. fragilis* were detected among the four groups in our dataset (**Figure 4C**) and this inconsistency remains to be further investigated. Furthermore, the genus *Bacteroides* and its representative species *B. ovatus* have been identified to have reduced abundance with colorectal tumor development (Zackular et al., 2014; Feng et al., 2015). *Bacteroides* species and their metabolic products, such as bile acid, have been demonstrated to promote liver carcinoma through the mechanism of DNA damage (Hagio, 2011). According to our data, this genus was most enriched in group C. The abundance of *Bacteroides* was reduced in both group I and group D, and *Bacteroides* were not found group IO, indicating that both anti-cancer methods reduced the abundance of this microbial genus and that surgery is more effective for eliminating the growth of *B. ovatus*. Although some strains of *E. coli* have been shown to participate in tumor growth in a previous study (Arthur and Jobin, 2012) and the genus was in low relative abundance in our data set, *Enterobacteriaceae* are commensal bacteria in human. Thus, the identification of this family as a potential biomarker for the IO group (**Figure 6**) may imply that surgery may not eradicate this family of microorganisms. Similar to previous descriptions (Atarashi et al., 2011; Kostic et al., 2012), we identified the genera *Clostridium*, family *Lachnospiraceae* and order *Clostridiales* as potential biomarkers for healthy individuals (**Figure 6**), indicating their important roles in the healthy human intestine. Taken together, these results raised an intriguing question regarding whether the validity of this metagenomic study could be confirmed and indicated that the novel roles of these previously identified microorganisms in CRC patients treated by surgery or chemotherapy require further investigated.

### Additional Bacterial Genera Associated With Surgery and Chemotherapy May Serve as Candidates to Improve Anti-cancer Treatments

A recent discovery has demonstrated that *F. nucleatum* can induce chemoresistance of colorectal tumors by modulating autophagy pathways (Yu T. et al., 2017). In general, *F. nucleatum* promotes CRC chemoresistance by activating autophagosome formation and subsequently inducing autophagy-related proteins, such as pULK1, ULK1, and ATG7, in colorectal tumor cells (Yu T. et al., 2017). This study also recommended that post- detection of *F. nucleatum* abundance may be an effective approach to predict the occurrence of chemoresistance. In our study, *F. nucleatum* was not detected at high abundance across the four sampling groups, suggesting that this species may not be the only indicator of chemoresistance-mediated CRC recurrence. Although the microbial diversity was largely reduced after surgery, additional biomarkers associated with post-surgery were identified (**Figure 6**), providing new candidates for mechanistic study and clinical diagnosis. However, identified potential bio-markers restricted to taxons above bacteria families and orders, thus following shotgun metagenomic approach is required to confirm bacterial identity on species level. Moreover, severe side effects are associated with current chemotherapy protocols (Vanesa et al., 2015). Increasing evidence implies that the gut microbiota provides valuable targets for the alleviation of chemotherapeutic side effect (Mármol et al., 2017). In this study, an in-depth 16s RNA analysis between CRC patients before or after chemotherapy was conducted. The putative biomarkers identified in this study, such as *V. dispar* and the genus *Sutterella* (**Figure 6**), may provide additional targets to enhance the dosage response to conventional chemotherapeutic cocktails and simultaneously reduce side effects. In particular, the genera *Sutterella*, was found to be enriched in pancreatic cancer patients in comparison to control group (Half et al., 2015). Although *V. dispar* has been recorded as infectious bacterial species for human (Bhatti and Frank, 2000), there is no direct link reported between this species and cancer. Thus, further investigations are necessary to characterize the correlations between these two potential biomarkers and CRC patients.

### Perspectives for the Improvement of Anti-CRC Treatments by Studying Commensal Microorganisms

Large variations of microbial structures between different populations and areas have been mentioned in previous studies (Yu J. et al., 2017). Thus, a larger scale analysis is required to obtain universal indicators for anti-cancer treatments, such as chemotherapy and surgery. In addition, patients can be grouped according to various physiological parameters, such as sex, age or BMI, to study the relationships between these factors and commensal microbiota. Moreover, microbiota differentiation can be observed in each individual CRC patient. Therefore, personalized diagnosis and suitable therapeutic methods will improve the quality of life and survival rate of CRC patients in the future. Interestingly, a comprehensive analysis has been developed along with the development of modern -omics technology. For example, metagenomic sequencing and microarray-based approaches may enhance the understanding of the functional relationships among commensal bacteria and CRC treatments (Hua et al., 2015). Meta-transcriptomic and proteomic studies that correlate the steady-state transcript and protein levels of the gut microbiota are considered to be powerful -omics tools for molecular profiling (Zhang et al., 2017). Additionally, expertise on bacterial strain isolation, culturing and molecular analysis are crucial for determining the anti-cancer properties of certain bacterial strains.

## Conclusion

The results of this study suggest that a general effect of the presence or absence of certain bacterial groups likely plays a pivotal role in anti-cancer treatments in CRC patients. Observations of species-specific variations in the microbiota from CRC patients may lead to the development and optimization of existing surgical or chemotherapeutic protocols. Therefore, unraveling the underlying mechanisms of this phenomenon is a priority for addressing how certain microbial strains can be affected after anti-cancer therapeutic treatments to cure CRC in the near future.

## Materials and Methods

### Group Information of CRC Patients

In total, stool samples from 69 individuals divided into four groups were collected at from the Peking University Shenzhen Hospital, China. In detail, 33 samples were from healthy individuals, denoted as group C; 17 samples were from CRC patients before treatment, denoted as group I; 5 samples were from CRC patients who were surgically treated, denoted as group IO; and 14 samples were from CRC patients who were chemotherapeutically treated, denoted as group D. The basic information of these subjects is listed in **Table 1**. The average age and BMI of patients of each clinical group were approximately 50 and 22, respectively. This study was approved by the Ethics Committee of the Peking University Shenzhen Hospital, China. Informed consent was obtained from all of the subjects in this study. For group D patients, a chemotherapeutic cocktail containing tegafur (precursor of 5′-fluorouracil, 40 mg/m^2^ bid po d1–d14) and oxaliplatin (130 mg/m^2^ ivgtt 2h d1) was applied for 21 days/cycle, with 6–8 cycles per treatment.

### Sampling Condition and Genomic DNA Extraction

Stool samples were taken and kept on dry ice before DNA extraction. DNA was extracted using the GenElute^TM^ Stool DNA Isolation Kit (Sigma-Aldrich, St. Louis, MO, United States) according to the manufacturer’s protocol. In general, approximately 200 mg stool samples were extracted by adding lysis buffer A and additive A in bead tubes. After vortexing for approximately 3–5 min, the lysate was transferred to a new 1.5 ml tube supplemented with binding buffer I on ice for 10 min. After a short spin, the resulting lysate was transferred into a new 1.5 ml tube with the addition of absolute ethanol for precipitation. Precipitated DNA was then loaded onto a binding column, and the eluted solution was discarded. Wash solution A was used to wash bound DNA twice. Distilled water was used to elute DNA. Subsequently, the DNA integrity, concentration and purity were monitored on 1% agarose gels (**Supplementary Figure S3**).

### Amplicon Generation and PCR Product Evaluation

16S rRNA genes of distinct regions (e.g., V4–V5) were amplified using specific primers (e.g., 16S: 338F and 806R/515F and 806R/515F and 907R) with a 12-bp barcode (**Supplementary Figures S4, S5**). The primers were synthesized by Invitrogen (Carlsbad, CA, United States). PCR, containing 25 μl of 2x Premix Taq (Takara Biotechnology, Dalian Co. Ltd., China), 1 μl of each primer (10 mM) and 3 μl of DNA (20 ng/μl) template in a total volume of 50 μl, was performed by thermocycling: 5 min at 94°C for initialization; 30 cycles of 30 s denaturation at 94°C, 30 s annealing at 52°C, and 30 s extension at 72°C; followed by 10 min final elongation at 72°C. Three replicates per sample and each PCR product of the same sample were mixed, and a BioRad S1000 thermocycler (Bio-Rad Laboratory, Hercules, CA, United States) was used.

The length and concentration of the PCR products were detected by 1% agarose gel electrophoresis. Samples with a bright main strip (e.g., 16S V4/V5: 400–450 bp) were used for further experiments. PCR products were mixed at equi-density ratios according to the GeneTool Analysis Software (Version4.03.05.0, SynGene). Then, the mixed PCR products were purified with the EZNA Gel Extraction Kit (Omega, United States).

### Library Preparation and NGS Sequencing

Sequencing libraries were generated using the NEBNext^®^ Ultra^TM^ DNA Library Prep Kit for Illumina^®^ sequencing (New England Biolabs, United States) following the manufacturer’s recommendations, and index codes were added. The library quality was assessed on a Qubit@ 2.0 Fluorometer (Thermo Scientific) and Agilent Bioanalyzer 2100 system. Finally, the library was sequenced on an Illumina Hiseq2500 platform, and 250 bp paired-end reads were generated.

### Quality Control Steps of the Raw NGS Sequencing Data

Quality filtering of the paired-end raw reads was performed under specific filtering conditions to obtain high-quality clean reads according to the Trimmomatic (V0.33)^1^ quality-control process. At the same time, sequences were assigned to each sample based on their unique barcode and primer, after which the barcodes and primers were removed and paired-end clean reads were recovered.

Paired-end clean reads were merged using FLASH (V1.2.11)^2^ (Magoč and Salzberg, 2011) according to the relationship of the overlap between the paired-end reads when at least 10 of the reads overlapped the read generated from the opposite end of the same DNA fragment, the maximum allowable error ratio of an overlap region of 0.2, and the spliced sequences were called raw tags.

Quality filtering on the spliced sequences was performed using Trimmomatic software (Bolger et al., 2014) to obtain effective clean tags.

### Identification and Quantification of Operational Taxonomic Units

Operational taxonomic units (OTU) clustering and species annotation were performed by USEARCH software (V8.0.1517)^3^ (Edgar, 2010). Sequences with ≥97% similarity were assigned to the same OTU. Representative sequences for each OTU were screened for further annotation. It is generally believed that a singleton OTU is obtained due to sequencing errors or chimeras generated during PCR. Therefore, singleton OTUs were removed using usearch^4^ after OTU clustering, and then, chimeric sequences were detected and removed using the UCHIME *de novo* algorithm^5^ (Edgar et al., 2011).

For each representative sequence, the GreenGene database (version gg_13_8)^6^ was used based on the RDP classifier algorithm (ucluster approach with default settings) and the assign_taxonomy.py script^7^ in QIIME (version 1.9.0)^8^ (Caporaso et al., 2010) to annotate the taxonomic information (set the confidence threshold to default to 0.5 or above). The polluted OTU and its Tags, which are annotated as chloroplasts or mitochondria (16S amplicons) and cannot be annotated at the kingdom level, were removed. Then, the number of effective tags (No. of seqs) and OTU taxonomic synthesis information were input into a table (otu_table) for the final analysis.

To study the phylogenetic relationship of different OTUs, KRONA software^9^ (Phillippy et al., 2011) was used to visualize the results of individual sample annotations. To quickly and intuitively study the species composition and abundance information in a sample, the GraPhlAn software^10^ (Asnicar et al., 2015) was used to obtain a single sample OTU annotation circle graph.

To study the difference in dominant species in different samples (groups), an OTU representative sequence with a relative abundance in the first 50 and annotated to the level of the genus was selected, multiple sequence alignments were conducted using FastTree software, and the relative abundance of each OTU and species annotation information of the representative sequence were combined with the ggtree software package for visual display.

Operational taxonomic unit abundance information was normalized using a standard of the sequence number corresponding to the sample with the least sequences. Subsequent analyses of the alpha diversity and beta diversity were performed basing on this output normalized data.

### Analysis of Alpha and Beta Diversity

Alpha diversity was applied to analyze the complexity of species diversity for a sample via 2 indices, including the Chao1 and Shannon and Simpson dominance. All of the indices in our samples were calculated with QIIME (V1.9.1) and displayed using R software (V2.15.3). Two indices were selected to identify community richness: the observed specie, and Chao1. Three indices were used to identify community diversity: Shannon, Simpson, and Dominance.

Beta diversity analysis was used to evaluate differences in species complexity in the samples, and beta diversity on both the weighted and unweighted unifrac were calculated by QIIME software. Principal coordinate analysis (PCoA) was performed to obtain the principal coordinates and visualize the species using complex, multidimensional data. A weighted or unweighted unifrac distance matrix among samples obtained previously was transformed to a new set of orthogonal axes, by which the maximum variation factor was demonstrated according to the first principal coordinate, the second maximum by the second principal coordinate, and so on. PCoA was performed by QIIME2 and the ggplot2 package in R software. Sample cluster analysis was performed using the UPGMA (Unweighted Pair-group Method with Arithmetic Means) method to interpret the distance matrix using the average linkages and was conducted by the upgma_cluster.py script^11^ in QIIME software. NMDS analysis was performed by the vegan package of R software based on the normalized OTU abundance table. Network analysis was performed by using bacterial genera with relative abundance higher than 0.5%. The network map was drawn by selecting Spearman correlations between each two bacterial groups (*P* < 0.05, *R* > 0.8).

### Statistical Analysis

PERMANOVA was performed among four groups of patients to determine variations between the microbial community structures. All the four groups shown significant differences in microbial structures (*P* = 0.001).

## Data Submission

The raw data used in this study were uploaded to Sequence Read Archive (https://www.ncbi.nlm.nih.gov/sra) under Bioproject PRJNA464414.

## Author Contributions

GLY designed the experiments. ZL and GLI performed the experiments. BL and XJ analyzed the data. XD wrote the manuscript. GLY critically commented and revised it.

## Conflict of Interest Statement

The authors declare that the research was conducted in the absence of any commercial or financial relationships that could be construed as a potential conflict of interest. The reviewer MW and handling editor declared their shared affiliation at time of review.

## Footnotes

1 http://www.usadellab.org/cms/?page=trimmomatic

2 https://ccb.jhu.edu/software/FLASH/

3 http://www.drive5.com/usearch/

4 http://www.drive5.com/usearch/manual/chimera_formation.html

5 http://www.drive5.com/usearch/manual/uchime_algo.html

6 http://greengenes.secondgenome.com/

7 http://qiime.org/scripts/assign_taxonomy.html

8 http://qiime.org/

9 http://sourceforge.net/projects/krona/

10 http://huttenhower.sph.harvard.edu/graphlan

11 http://qiime.org/scripts/upgma_cluster.html

## Supplementary Material

FIGURE S1 Schematic view of the experimental design and analytical pipeline of this study. CRC, colorectal cancer; OTUs, operational taxonomic units.

FIGURE S2 Evaluation of sample preparation and sequencing quality. **(A)** OTU dilution curve, **(B)** Shannon curve, and **(C)** OTU rank abundance.

FIGURE S3 Heatmap representation of microbial species that were significantly different among group comparisons.

FIGURE S4 Quality of extracted DNA.

FIGURE S5 DNA gel of V4-V5 amplicon.

TABLE S1 Alpha-diversity indexes of each sample.

## References

[B1] Abed J. Emgård J. E. M. Zamir G. Faroja M. Almogy G. Grenov A. (2016). Fap2 mediates *Fusobacterium nucleatum* colorectal adenocarcinoma enrichment by binding to tumor-expressed Gal-GalNAc. *Cell Host Microbe* 20 215–225. 10.1016/j.chom.2016.07.006 27512904 PMC5465824

[B2] Arthur J. C. Jobin C. (2012). Intestinal inflammation targets cancer-inducing activity of the microbiota. *Science* 338 120–123. 10.1126/science.1224820 22903521 PMC3645302

[B3] Asnicar F. Weingart G. Tickle T. L. Huttenhower C. Segata N. (2015). Compact graphical representation of phylogenetic data and metadata with GraPhlAn. *PeerJ* 3:e1029. 10.7717/peerj.1029 26157614 PMC4476132

[B4] Atarashi K. Tanoue T. Shima T. Imaoka A. Kuwahara T. Momose Y. (2011). Induction of colonic regulatory T cells by indigenous *Clostridium* species. *Science* 331 337–341. 10.1126/science.1198469 21205640 PMC3969237

[B5] Bernard Levin M. D. Lieberman D. A. Beth Mcfarl M. D. Smith R. A. Durado Brooks M. D. Andrews M. K. S. (2008). Screening and surveillance for the early detection of colorectal cancer and adenomatous polyps, 2008: a joint guideline from the American cancer society, the us multi-society task force on colorectal cancer, and the American college of radiology. *CA Cancer J. Clin.* 58 130–160. 10.3322/CA.2007.0018 18322143

[B6] Bhatti M. A. Frank M. O. (2000). *Veillonella parvula* meningitis: case report and review of *Veillonella* infections. *Clin. Infect. Dis.* 31 839–840. 10.1086/314046 11017846

[B7] Boleij A. Schaeps R. M. Tjalsma H. (2009). Association between *Streptococcus bovis* and colon cancer. *J. Clin. Microbiol.* 47:516. 10.1128/JCM.01755-08 19189926 PMC2643683

[B8] Bolger A. M. Lohse M. Usadel B. (2014). Trimmomatic: a flexible trimmer for Illumina sequence data. *Bioinformatics* 30 2114–2120. 10.1093/bioinformatics/btu170 24695404 PMC4103590

[B9] Boon N. Windt W. D. Verstraete W. Top E. M. (2002). Evaluation of nested PCR–DGGE (denaturing gradient gel electrophoresis) with group-specific 16S rRNA primers for the analysis of bacterial communities from different wastewater treatment plants. *FEMS Microbiol. Ecol.* 39 101–112. 10.1111/j.1574-6941.2002.tb00911.x 19709189

[B10] Brenner H. Kloor M. Pox C. P. (2014). Colorectal cancer. *Lancet* 383 1490–1502. 10.1016/S0140-6736(13)61649-9 24225001

[B11] Caporaso J. G. Kuczynski J. Stombaugh J. Bittinger K. Bushman F. D. Costello E. K. (2010). QIIME allows analysis of high-throughput community sequencing data. *Nat. Methods* 7 335–336. 10.1038/nmeth.f.303 20383131 PMC3156573

[B12] Cartwright T. H. (2012). Treatment decisions after diagnosis of metastatic colorectal cancer. *Clin. Colorectal Cancer* 11 155–166. 10.1016/j.clcc.2011.11.001 22192364

[B13] Castellarin M. Warren R. L. Freeman J. D. Dreolini L. Krzywinski M. Strauss J. (2012). *Fusobacterium nucleatum* infection is prevalent in human colorectal carcinoma. *Genome Res.* 22 299–306. 10.1101/gr.126516.111 22009989 PMC3266037

[B14] Cheesman S. E. Neal J. T. Mittge E. Seredick B. M. Guillemin K. (2011). Epithelial cell proliferation in the developing zebrafish intestine is regulated by the Wnt pathway and microbial signaling via Myd88. *Proc. Natl. Acad. Sci. U.S.A.* 108(Suppl. 1) 4570–4577. 10.1073/pnas.1000072107 20921418 PMC3063593

[B15] Chen H. M. Yu Y. N. Wang J. L. Lin Y. W. Kong X. Yang C. Q. (2013). Decreased dietary fiber intake and structural alteration of gut microbiota in patients with advanced colorectal adenoma. *Am. J. Clin. Nutr.* 97 1044–1052. 10.3945/ajcn.112.046607 23553152

[B16] Chen W. Liu F. Ling Z. Tong X. Xiang C. (2012). Human intestinal lumen and mucosa-associated microbiota in patients with colorectal cancer. *PLoS One* 7:e39743. 10.1371/journal.pone.0039743 22761885 PMC3386193

[B17] Clemente J. C. Ursell L. K. Parfrey L. W. Knight R. (2012). The impact of the gut microbiota on human health: an integrative view. *Cell* 148 1258–1270. 10.1016/j.cell.2012.01.035 22424233 PMC5050011

[B18] Costello E. K. Lauber C. L. Hamady M. Fierer N. Gordon J. I. Knight R. (2009). Bacterial community variation in human body habitats across space and time. *Science* 326 1694–1697. 10.1126/science.1177486 19892944 PMC3602444

[B19] Dahan L. Sadok A. Formento J. L. Seitz J. F. Kovacic H. (2009). Modulation of cellular redox state underlies antagonism between oxaliplatin and cetuximab in human colorectal cancer cell lines. *Br. J. Pharmacol.* 158 610–620. 10.1111/j.1476-5381.2009.00341.x 19732064 PMC2757701

[B20] Dolara P. Caderni G. Salvadori M. Morozzi G. Fabiani R. Cresci A. (2002). Fecal levels of short-chain fatty acids and bile acids as determinants of colonic mucosal cell proliferation in humans. *Nutr. Cancer* 42 186–190. 10.1207/S15327914NC422_;6 12416258

[B21] Dove W. F. Clipson L. Gould K. A. Luongo C. Marshall D. J. Moser A. R. (1997). Intestinal neoplasia in the ApcMin mouse: independence from the microbial and natural killer (beige locus) status. *Cancer Res.* 57 812–814. 9041176

[B22] Edgar R. C. (2010). Search and clustering orders of magnitude faster than BLAST. *Bioinformatics* 26 2460–2461. 10.1093/bioinformatics/btq461 20709691

[B23] Edgar R. C. Haas B. J. Clemente J. C. Quince C. Knight R. (2011). UCHIME improves sensitivity and speed of chimera detection. *Bioinformatics* 27 2194–2200. 10.1093/bioinformatics/btr381 21700674 PMC3150044

[B24] Fearon E. R. (2011). Molecular genetics of colorectal cancer. *Annu. Rev. Pathol.* 6 479–507. 10.1146/annurev-pathol-011110-130235 21090969

[B25] Feng Q. Liang S. Jia H. Stadlmayr A. Tang L. Lan Z. (2015). Gut microbiome development along the colorectal adenoma–carcinoma sequence. *Nat. Commun.* 6:6528. 10.1038/ncomms7528 25758642

[B26] Ferlay J. Soerjomataram I. Ervik M. Dikshit R. Eser S. Mathers C. (2013). *GLOBOCAN 2012 v1.0 Cancer Incidence and Mortality Worldwide*. Lyon: International Agency for Research on Cancer. 10.1002/ijc.29210 25220842

[B27] Geng J. Hong F. Tang X. Zhai H. Zhang Z. (2013). Diversified pattern of the human colorectal cancer microbiome. *Gut Pathog.* 5:2. 10.1186/1757-4749-5-2 23497613 PMC3599420

[B28] Hagio M. (2011). Bile acid is a host factor that regulates the composition of the cecal microbiota in rats. *Gastroenterology* 141 1773–1781. 10.1053/j.gastro.2011.07.046 21839040

[B29] Half E. Keren N. Dorfman T. Reshef L. Lachter I. Kluger Y. (2015). P-165Specific changes in fecal microbiota may differentiate Pancreatic Cancer patients from healthy individuals. *Ann. Oncol.* 26:iv48 10.1093/annonc/mdv233.165

[B30] Hooper L. V. Gordon J. I. (2001). Commensal host–bacterial relationship. *Science* 292 1115–1118. 10.1126/science.1058709 11352068

[B31] Hua Z. S. Han Y. J. Chen L. X. Liu J. Hu M. Li S. J. (2015). Ecological roles of dominant and rare prokaryotes in acid mine drainage revealed by metagenomics and metatranscriptomics. *ISME J.* 9 1280–1294. 10.1038/ismej.2014.212 25361395 PMC4438317

[B32] Huxley R. R. Ansary-Moghaddam A. Clifton P. Parr C. L. Czernichow S. Woodward M. (2009). The impact of dietary and lifestyle risk factors on risk of colorectal cancer: a quantitative overview of the epidemiological evidence. *Int. J. Cancer* 125 171–180. 10.1002/ijc.24343 19350627

[B33] Ichinohe T. Pang I. K. Kumamoto Y. Peaper D. R. Ho J. H. Murray T. S. (2011). Microbiota regulates immune defense against respiratory tract influenza A virus infection. *Proc. Natl. Acad. Sci. U.S.A.* 108 5354–5359. 10.1073/pnas.1019378108 21402903 PMC3069176

[B34] Iida N. Dzutsev A. Stewart C. A. Smith L. Bouladoux N. Weingarten R. A. (2013). Commensal bacteria control cancer response to therapy by modulating the tumor microenvironment. *Science* 342 967–970. 10.1126/science.1240527 24264989 PMC6709532

[B35] Jemal A. (2011). Global cancer statistics. *CA Cancer J. Clin.* 61 134–134. 10.3322/caac.20107 21296855

[B36] Kelland L. (2007). The resurgence of platinum-based cancer chemotherapy. *Nat. Rev. Cancer* 7 573–584. 10.1038/nrc2167 17625587

[B37] Kostic A. D. Gevers D. Pedamallu C. S. Michaud M. Duke F. Earl A. M. (2012). Genomic analysis identifies association of Fusobacterium with colorectal carcinoma. *Genome Res.* 22 292–298. 10.1101/gr.126573.111 22009990 PMC3266036

[B38] Larsson S. C. Rafter J. Holmberg L. Bergkvist L. Wolk A. (2005). Red meat consumption and risk of cancers of the proximal colon, distal colon and rectum: the Swedish Mammography Cohort. *Int. J. Cancer* 113 829–834. 10.1002/ijc.20658 15499619

[B39] Lee Y. K. Mazmanian S. K. (2010). Has the microbiota played a critical role in the evolution of the adaptive immune system? *Science* 330 1768–1773. 10.1126/science.1195568 21205662 PMC3159383

[B40] Magoč T. Salzberg S. L. (2011). FLASH: fast length adjustment of short reads to improve genome assemblies. *Bioinformatics* 27 2957–2963. 10.1093/bioinformatics/btr507 21903629 PMC3198573

[B41] Mármol I. Sánchezdediego C. Dieste A. P. Cerrada E. (2017). Colorectal carcinoma: a general overview and future perspectives in colorectal cancer. *Int. J. Mol. Sci.* 18:E197. 10.3390/ijms18010197 28106826 PMC5297828

[B42] McAleer J. P. Kolls J. K. (2012). Maintaining poise: commensal microbiota calibrate interferon responses. *Immunity* 37 10–12. 10.1016/j.immuni.2012.07.001 22840839

[B43] Nugent F. W. Haggitt R. C. Gilpin P. A. (2005). Cancer surveillance in ulcerative colitis. *Gastroenterology* 92 928–936. 2013371

[B44] Phillippy A. M. Bergman N. H. Ondov B. D. (2011). Interactive metagenomic visualization in a Web browser. *BMC Bioinformatics* 12:385. 10.1186/1471-2105-12-385 21961884 PMC3190407

[B45] Rakoff-Nahoum S. Medzhitov R. (2007). Regulation of spontaneous intestinal tumorigenesis through the adaptor protein MyD88. *Science* 317 124–127. 10.1126/science.1140488 17615359

[B46] Rubinstein M. R. Wang X. Liu W. Hao Y. Cai G. Han Y. W. (2013). *Fusobacterium nucleatum* promotes colorectal carcinogenesis by modulating E-Cadherin/β-Catenin signaling via its FadA adhesin. *Cell Host Microbe* 14 195–206. 10.1016/j.chom.2013.07.012 23954158 PMC3770529

[B47] Sears C. L. Islam S. Saha A. Arjumand M. Alam N. H. Faruque A. S. G. (2008). Association of Enterotoxigenic *Bacteroides fragilis* infection with inflammatory diarrhea. *Clin. Infect. Dis.* 47 797–803. 10.1086/591130 18680416 PMC3045827

[B48] Seder C. W. Kramer M. Long G. Uzieblo M. R. Shanley C. J. Bove P. (2009). *Clostridium septicum* aortitis: report of two cases and review of the literature. *J. Vasc. Surg.* 49 1304–1309. 10.1016/j.jvs.2008.11.058 19307090

[B49] Sobhani I. Tap J. Roudot-Thoraval F. Roperch J. P. Letulle S. Langella P. (2011). Microbial dysbiosis in colorectal cancer (CRC) patients. *PLoS One* 6:e16393. 10.1371/journal.pone.0016393 21297998 PMC3029306

[B50] Stappenbeck T. S. Hooper L. V. Gordon J. I. (2002). Developmental regulation of intestinal angiogenesis by indigenous microbes via Paneth cells. *Proc. Natl. Acad. Sci. U.S.A.* 99 15451–15455. 10.1073/pnas.202604299 12432102 PMC137737

[B51] Vanesa S. Samy S. Kulmira N. (2015). Platinum-based chemotherapy: gastrointestinal immunomodulation and enteric nervous system toxicity. *Am. J. Physiol. Gastrointest. Liver Physiol.* 308 G223–G232. 10.1152/ajpgi.00212.2014 25501548

[B52] Vogelstein B. Kinzler K. W. (2004). Cancer genes and the pathways they control. *Nat. Med.* 10 789–799. 10.1038/nm1087 15286780

[B53] Walko C. M. Lindley C. (2005). Capecitabine: a review. *Clin. Ther.* 27 23–44. 10.1016/j.clinthera.2005.01.005 15763604

[B54] Wang C. Li J. (2012). An update on chemotherapy of colorectal liver metastases. *World J. Gastroenterol.* 18 25–33. 10.3748/wjg.v18.i1.25 22228967 PMC3251802

[B55] Wang T. Cai G. Qiu Y. Fei N. Zhang M. Pang X. (2011). Structural segregation of gut microbiota between colorectal cancer patients and healthy volunteers. *ISME J.* 6 320–329. 10.1038/ismej.2011.109 21850056 PMC3260502

[B56] Yu J. Feng Q. Wong S. H. Zhang D. Liang Q. Y. Qin Y. (2017). Metagenomic analysis of faecal microbiome as a tool towards targeted non-invasive biomarkers for colorectal cancer. *Gut* 66 70–78. 10.1136/gutjnl-2015-309800 26408641

[B57] Yu T. Guo F. Yu Y. Sun T. Ma D. Han J. (2017). *Fusobacterium nucleatum* promotes chemoresistance to colorectal cancer by modulating autophagy. *Cell* 170 548–563. 10.1016/j.cell.2017.07.008 28753429 PMC5767127

[B58] Zackular J. P. Baxtera N. T. Iversona K. D. Sadlerb W. D. Petrosinoc J. F. Chen G. Y. (2013). The gut microbiome modulates colon tumorigenesis. *mBio* 4:e00692. 10.1128/mBio.00692-13 24194538 PMC3892781

[B59] Zackular J. P. Rogers M. A. Schloss P. D. (2014). The human gut microbiome as a screening tool for colorectal cancer. *Cancer Prev. Res.* 7 1112–1121. 10.1158/1940-6207.CAPR-14-0129 25104642 PMC4221363

[B60] Zhang X. Chen W. Ning Z. Mayne J. Mack D. Stintzi A. (2017). Deep metaproteomics approach for the study of human microbiomes. *Anal. Chem.* 89 9407–9415. 10.1021/acs.analchem.7b02224 28749657

[B61] Zou W. Wolchok J. D. Chen L. (2016). PD-L1 (B7-H1) and PD-1 pathway blockade for cancer therapy: mechanisms, response biomarkers and combinations. *Sci. Trans. Med.* 8:328rv324. 10.1126/scitranslmed.aad7118 26936508 PMC4859220

