# Supplementary figures and images for: Comparison of Microbiota in Patients Treated by Surgery or Chemotherapy by 16S rRNA Sequencing Reveals Potential Biomarkers for Colorectal Cancer Therapy
Source: Front Microbiol. 2018 Jul 17;9:1607. doi: 10.3389/fmicb.2018.01607 (PMC6057110; doi:10.3389/fmicb.2018.01607)

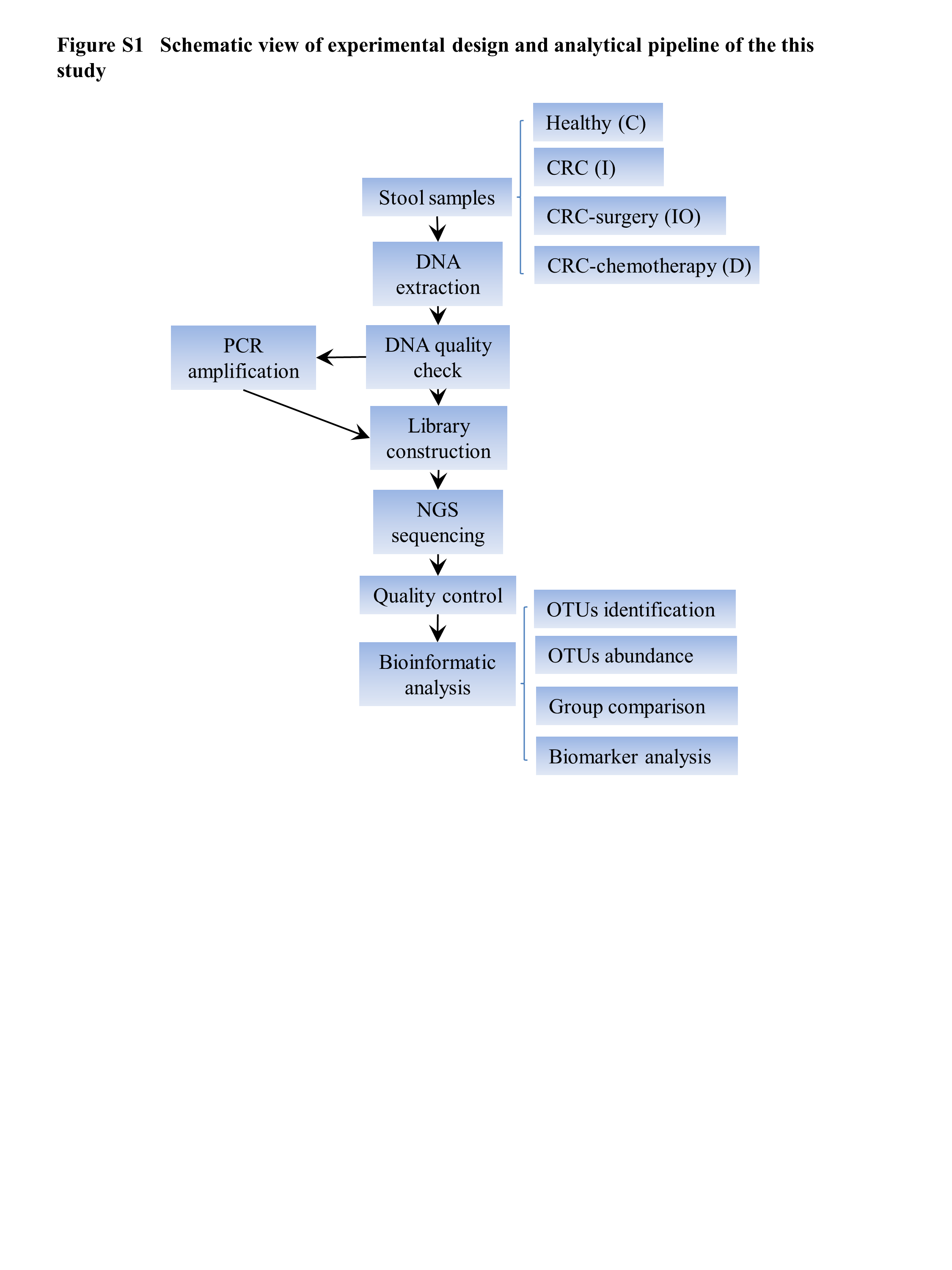

Supplement: FIGURE S1 — Schematic view of the experimental design and analytical pipeline of this study. CRC, colorectal cancer; OTUs, operational taxonomic units. [file Image_1.TIF]

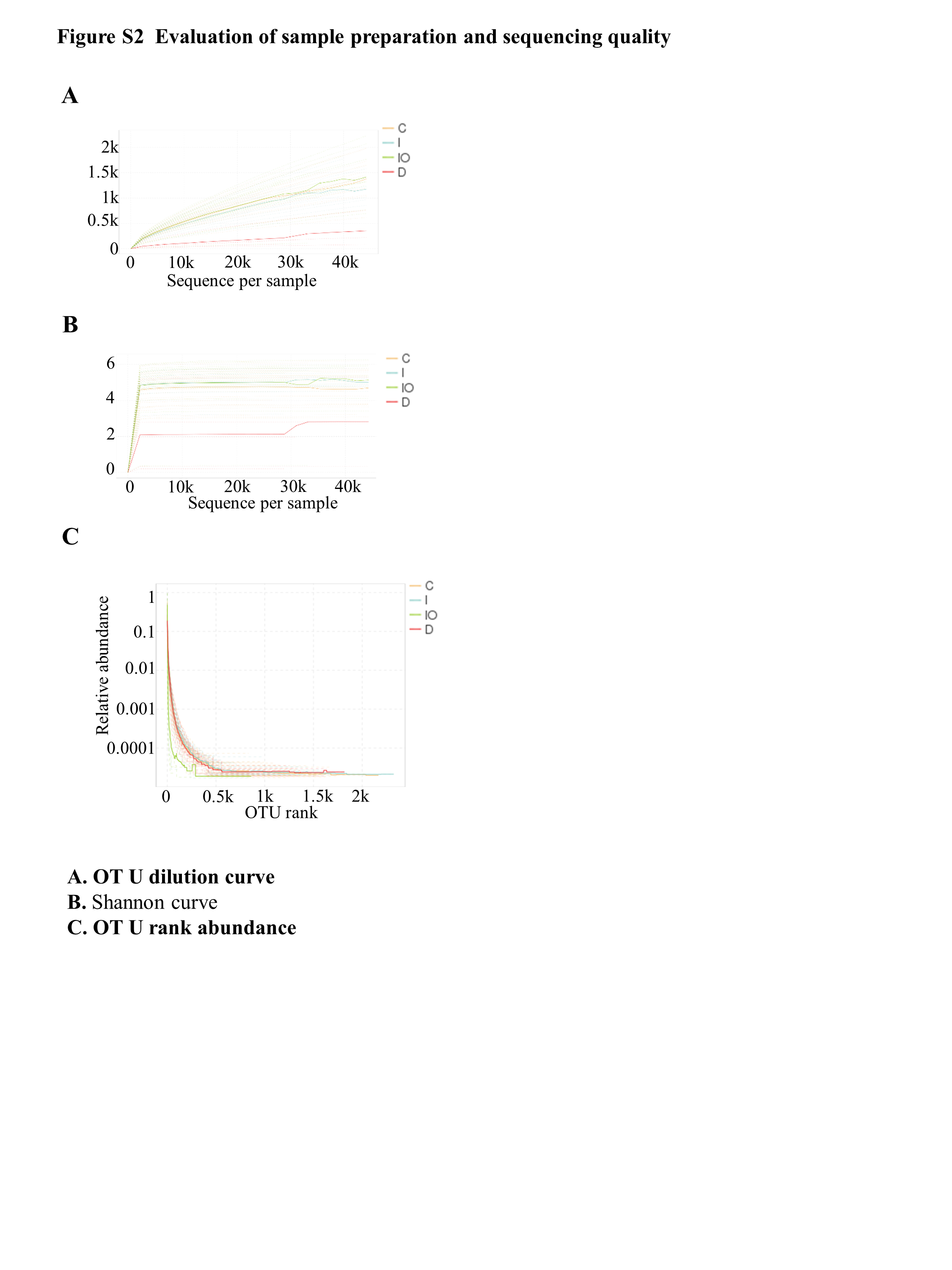

Supplement: FIGURE S2 — Evaluation of sample preparation and sequencing quality. (A) OTU dilution curve, (B) Shannon curve, and (C) OTU rank abundance. [file Image_2.TIF]

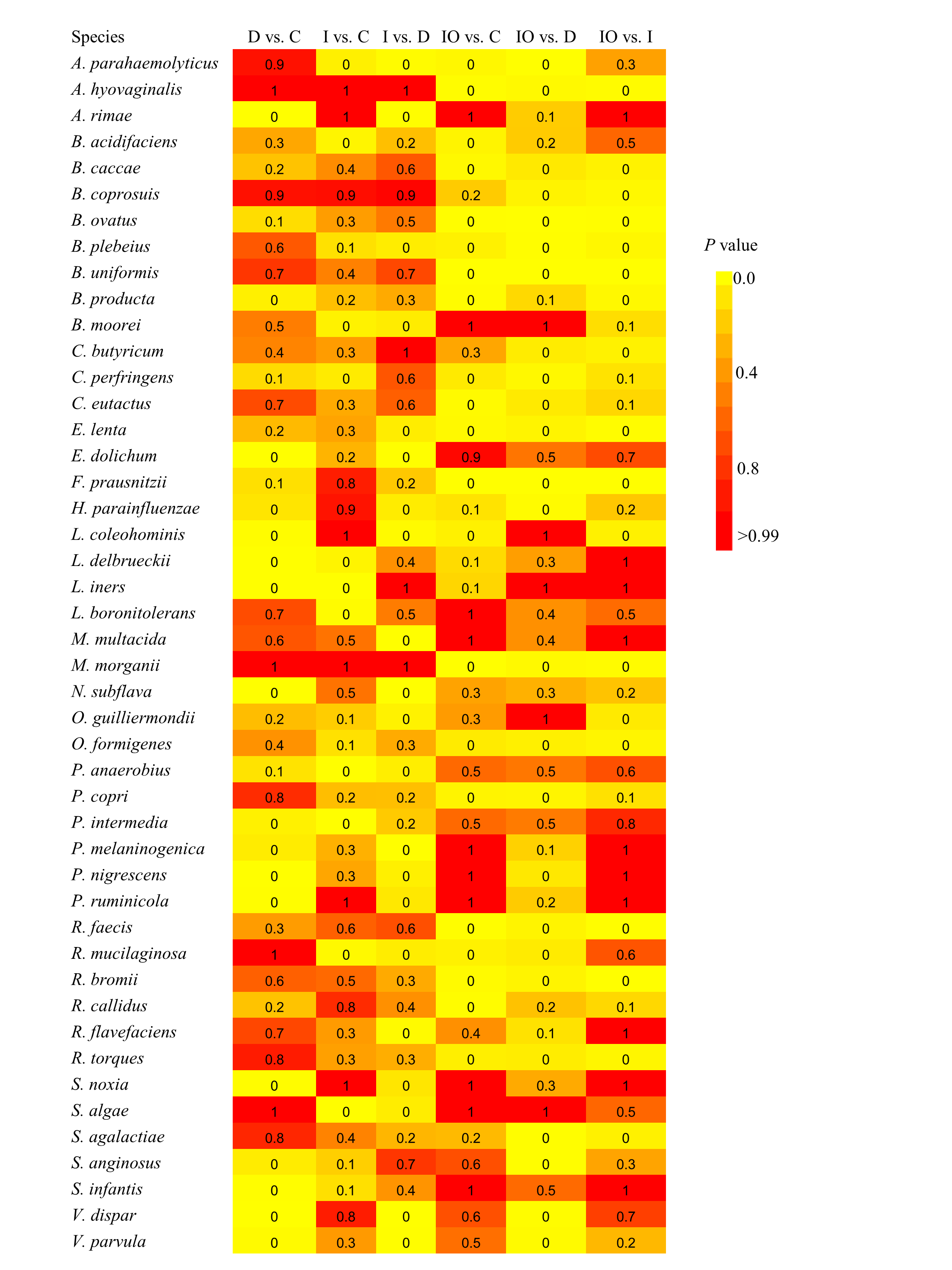

Supplement: FIGURE S3 — Heatmap representation of microbial species that were significantly different among group comparisons. [file Image_3.TIF]

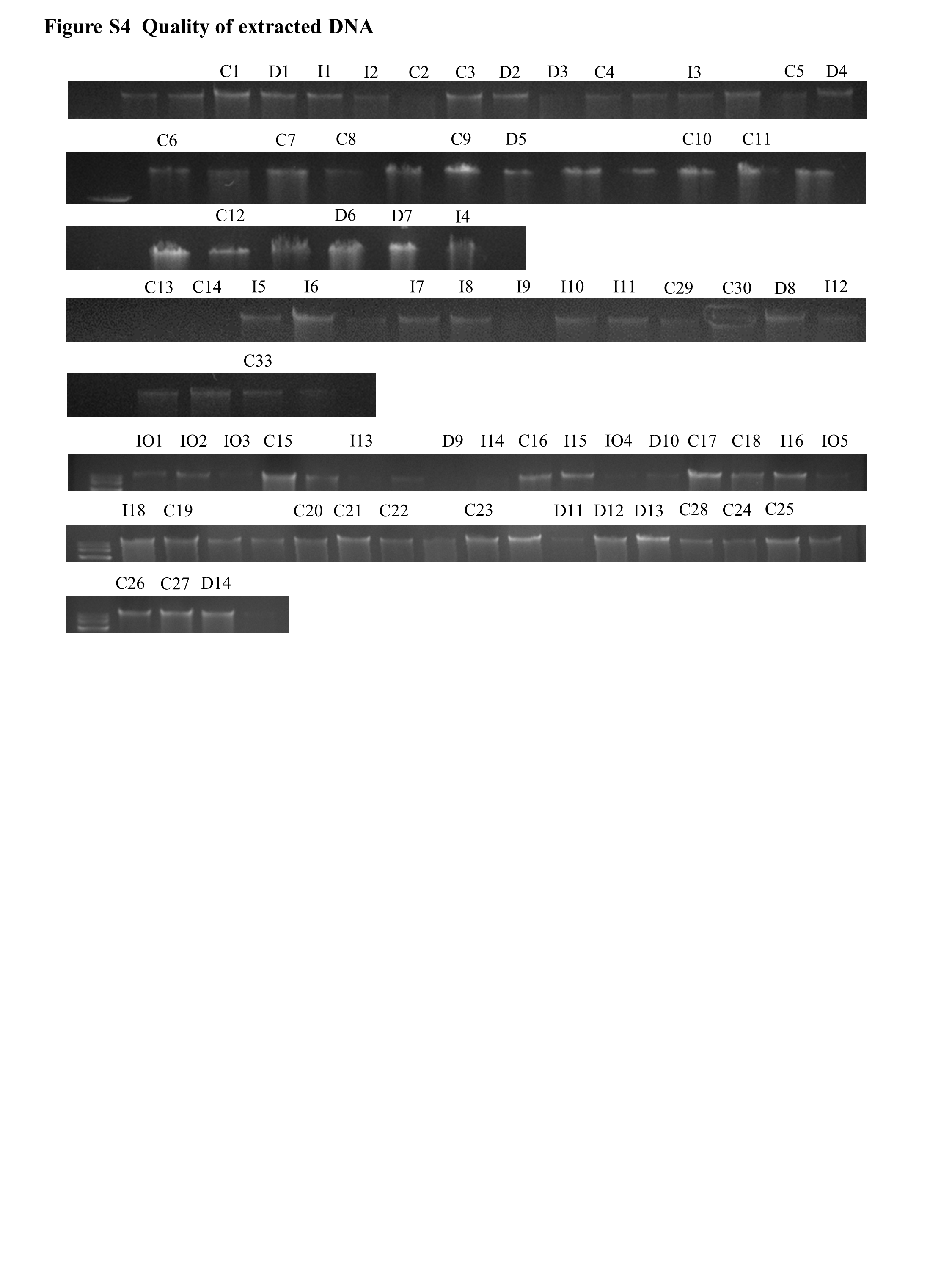

Supplement: FIGURE S4 — Quality of extracted DNA. [file Image_4.TIF]

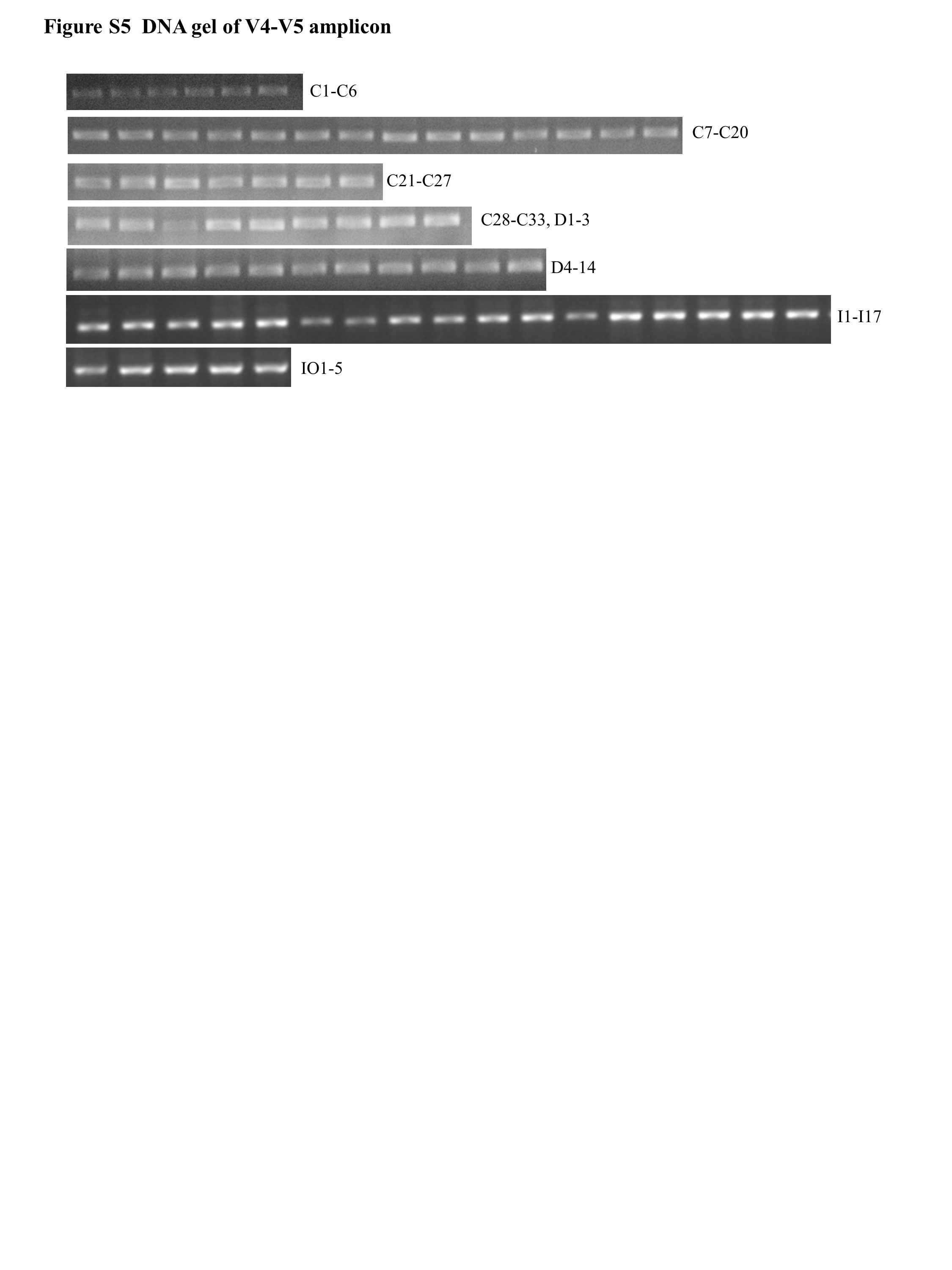

Supplement: FIGURE S5 — DNA gel of V4-V5 amplicon. [file Image_5.TIF]
